# Spatial-temporal variations of *Schistosoma japonicum* distribution after an integrated national control strategy: a cohort in a marshland area of China

**DOI:** 10.1186/1471-2458-13-297

**Published:** 2013-04-04

**Authors:** Yi-Biao Zhou, Song Liang, Geng-Xin Chen, Chris Rea, Shi-Min Han, Zong-Gui He, Yuan-Pei Li, Jian-Guo Wei, Gen-Ming Zhao, Qing-Wu Jiang

**Affiliations:** 1 Department of Epidemiology, School of Public Health, Fudan University, 138 Yixueyuan Road, Shanghai, 200032, China; 2 Key Laboratory of Public Health Safety, Fudan University, Ministry of Education, Shanghai, China; 3 Department of Environmental and Global Health, College of Public Health and Health Professions, University of Florida, Gainesville, FL, 32610, USA; 4 Emerging Pathogens Institute, University of Florida, Gainesville, FL, 32610, USA; 5 Guichi Anti-schistosomiasis Station, Anhui, China; 6 College of Public Health, The Ohio State University, Columbus, OH, USA

**Keywords:** Schistosomiasis, Intervention, Geographical information system, Spatial-temporal distribution, Spatial autocorrelation analysis, Spatial scan statistic, *Schistosoma japonicum*

## Abstract

**Background:**

Schistosomiasis transmission is typically focal. Understanding spatial variations of *Schistosoma* infections and their associated factors is important to help to invent site-specific intervention strategies.

**Methods:**

A five-year longitudinal study was carried out prospectively in 12 natural villages, Guichi district of Anhui province. A GIS-based spatial analysis was conducted to identify geographic distribution patterns of schistosomiasis infections at the household scale.

**Results:**

The results of the spatial autocorrelation analysis for 2005 showed that there were significant spatial clusters of human infections at the household level, and these results were in agreement with that of the spatial scan statistic. As prevalence of infections in humans decreased over the course of control, the spatial distribution of these infections became less heterogeneous.

**Conclusions:**

The findings imply that it may be necessary to re-assess risk factors of *S. japonicum* transmission over the course of control and to adjust accordingly control measures in the communities.

## Background

Schistosomiasis, a snail-mediated helminthiasis, is one of the most prevalent parasitic diseases in the world [1]. The disease is endemic in approximately 70 countries with about 200 million people affected worldwide [2-4]. Documented evidence indicates that *Schistosoma japonicum* has existed in China for over 2100 years [5]. Results of large-scale epidemiological surveys showed that schistosomiasis was distributed in 12 provinces and an estimated 11.6 million people were infected after the founding of the People’s Republic of China [6]. Over the past 50 years significant achievements have been made on schistosomiasis control through ongoing national control programs [7]. To date, five out of 12 formerly *S. japonicum*-endemic provinces have reached the national criteria of transmission interruption (i.e. no new infection), and the estimated number of human infections has been reduced from 1,638,103 in 1989 (the first nationwide schistosomiasis sampling survey) to 726,112 in 2004 (the third nationwide schistosomiasis sampling survey), reflecting a 55.7% decrease [8]. Despite these achievements, schistosomiasis is still of considerable economic and public health concern in China due to its potential for re-infection and recent reemergence in some previously controlled areas [5]. In 2004, two targets were established by the State Council of China – reduce the human prevalence of infection to less than 5% in all endemic counties by 2008, and then to < 1% by 2015 [9]. In order to achieve these goals, an integrated control strategy, aimed at reducing the roles of bovines and humans as sources of infection for snails, has been conducted in a number of endemic regions of China [9].

Given the extensively ongoing national control programs, an immediate question, which remains largely unaddressed, is how these control programs are changing local epidemiological characteristics of the disease and if the control programs are sufficient to suppress the transmission towards the set goals. To address the question, we need to first understand how local patterns of the disease change over the course of control; typically these patterns have both spatial and temporal dimensions. Advances in geographical information systems (GIS) and statistical methodologies for spatial analysis provide powerful tools to help characterize and improve our understanding of space-time distribution of diseases [10-16]. However, despite a large body of studies reporting spatial and/or temporal patterns of schistosomiasis infections, few studies have analyzed the space-time changes in the disease prevalence at a finer scale (e.g., below county level), especially before and after an integrated national control strategy [7,10-12,17,18]. In this study, GIS-based spatial analyses involving spatial autocorrelation analysis and spatial scan statistic were employed to identify geographic distribution patterns of schistosomiasis at the household level. Furthermore, changes in these patterns were evaluated over time in the Qiupu River region, where the integrated national control strategy was implemented. Knowledge on changes in the local epidemiological situation will aid in implementing and adjusting anti-schistosomiasis measures, and also increase the efficacy of these measures.

## Methods

### Study location

**Figure 1:**
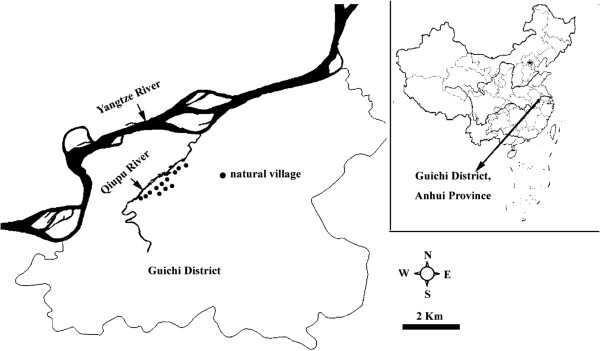
The study location in the Qiupu River region in Guichi district, Anhui province, People’s Republic of China.

The study took place in the Qiupu River region in Guichi district, Anhui province, China, which is, historically, one of the most serious areas of *S. japonicum* in China (Figure 1). This study region has a population of approximately 32,000 people and 5.7 km^2^ of snail-infected area [19]. Most of the residents are farmers living along the Qiupu River, and their principal activity is the cultivation of rice. The residents frequently contact infested water due to their working and living circumstances. *Oncomelania hupensis*, the intermediate snail host for *S. japonicum*, is distributed in the marshlands of the Qiupu River [19].

An integrated national control strategy was implemented in the Qiupu River watershed starting in 2006 [9,19]. Details on the interventions were reported in our previous work [19] and the main measures included: 1) all bovines were removed from the study area in 2006; 2) all residents aged 5-65 years were checked annually for schistosomiasis infection (detected annually using indirect hemagglutination assay (IHA) and all IHA positives were examined using Kato-Katz thick smear test (three slides) to confirm infection, except for those who received direct fecal examination) and only the residents with positive egg counts were treated with a single oral dose of praziquantel (40 mg/kg body weight); 3) the sanitation facilities were improved by constructing lavatories and latrines and tap water was supplied; 4) a program of health education was implemented focusing on avoidance of snail-infected areas and the associated river water; 5) some snail habitats were managed by agricultural methods (e.g. plant wheat).

### Cohort population

Twelve natural villages (i.e. a hamlet, which is a small group of dwellings under an administrative village which typically consists of natural villages) were selected randomly from our study region in 2005 and followed until 2009 (Figure 1). A cohort of population was selected from the 12 natural villages. The inclusion criteria for households were that: a) the household was from the selected natural village, b) the household resided in the natural village for more than 1 year, and c) the household had residents aged 5-65 years. The inclusion criteria for residents were that an individual must be: a) a resident of the selected household, b) 5-65 years of age, and c) no plan to migrate out of the natural village during the study period. Within the cohort, each observed resident was georeferenced by household. Georeferencing was implemented using a hand-held global positioning system (GPS) (Mobile Mapper TM, Thales Navigation, Inc., USA). Each resident of the cohort had his/her stool (a fecal specimen each individual) examined annually during the autumn season using the microscopic, quantitative Kato-Katz thick smear (3 slides) technique [18], and the detail of this stool examination was reported by Zhou et al. 2011 [20]. The outcome measure was incidence for the defined cohort of population except for 2005 (a baseline survey, and the measure was infection prevalence). Residents with a positive fecal examination result were treated with a single dose of praziquantel (40 mg/kg).

Written informed consent was obtained from all adult participants and from the parents or legal guardians of children aged < 18 years. Ethical approval for the study was endorsed by the Ethical Review Committee of School of Public Health, Fudan University.

### Data manipulation and statistical analysis

Data were entered in Microsoft Excel version 2003 (Microsoft Corp., Redmond, Wash.). A human *S. japonicum* infection was defined as the presence of at least one egg in three Kato-Katz smears.

The cohort characteristics and infection prevalence/incidence were analyzed using the SPSS Statistical Package for Social Sciences (SPSS Inc., Chicago, IL, 2007). Human prevalencewas analyzed using a generalized linear model (GLM) with a logit link and a binomial distribution [21,22]. The base model included year (from 2005 to 2009), sex, and age.

### Spatial autocorrelation analysis

Both Global and Local spatial autocorrelation analyses were implemented in OpenGeoda 0.9.8.14 software (http://geodacenter.asu.edu/software). Spatial autocorrelation analysis was employed to detect significant differences from a random spatial distribution of schistosomiasis. Moran’s I spatial autocorrelation statistics were calculated and visualized in the form of Moran Scatter Plots. In order to take into account variance instability of infection prevalences over time in both Global and Local spatial autocorrelation analyses, the empirical Bayes (EB) adjustment for the variance instability of prevalences was used [14]. The spatial weight files were created by using Distance-Band weight. A significance assessment was performed by means of a permutation test, and a reference distribution was generated under the assumption that the prevalence was randomly distributed. The number of permutation tests was set to 9999 and the significant *P*-level was set as 0.05.

### Spatial cluster analysis

Spatial analyses were performed in the SaTScan™ v8.2.1 software (Kulldorff M. and Information Management Services, Inc.). Spatial scan statistics were used to detect and evaluate the clusters of schistosomiasis cases. These were done by gradually scanning a circle window across space and noting the number of observed and expected observations inside the window at each setting. The Bernoulli model [15,16] for high prevalences was used, and the maximum spatial cluster size was set to 50% percent of the population at risk. Cases were defined as the population infected with *S. japonicum*, and controls were the remainder of the population. Monte Carlo hypothesis testing [23] was used in this study and the number of replications was set to 9999 with significance at the 0.05 level.

### GIS mapping

A digitized polygon map was obtained for the Qiupu River watershed at a scale of 1:50,000. Clusters were mapped using ArcGIS 9.2 software (Environmental Systems Research Institute, Inc., Redlands, CA, USA) in order to identify their physical location.

## Results

### Cohort characteristics

The population characteristics of the observed cohort are shown in Table 1. The mean age of the population increased by year, except for 2009 (*F* = 6.15, *P* =0.000).

**Table 1:** Characteristics of the cohort population examined from 2005 to 2009

**Year**	**Number of households**	**Mean of households per natural village**	**Number of population examined**	**Mean of population per natural village**	**Mean of residents per household**	**Mean age (year)**
2005	275	22.9	654	55	2.38	37.4
		(16-35)		(33-85)		(6.7-65.0)
						[36.2-38.6]
2006	277	23.1	652	54	2.35	38.2
		(16-35)		(33-84)		(7.7-64.5)
						[37.1-39.4]
2007	275	22.9	649	54	2.36	40.2
		(16-35)		(33-83)		(9.7-65.0)
						[39.0-41.4]
2008	261	21.8	575	48	2.20	41.2
		(12-32)		(25-85)		(7.3-65.0)
						[39.9-42.4]
2009	268	22.3	684	57	2.55	38.2
		(13-35)		(27-85)		(5.9-65.0)
						[36.9-39.4]

The range min-max values in parentheses (), 95% CI in square bracket [].

### Infection prevalence and global spatial autocorrelation

**Figure 2:**
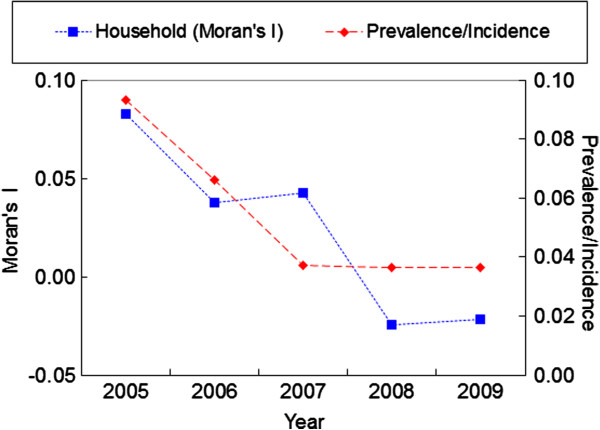
**Global spatial autocorrelation analyses for annual prevalence/incidence of infection *S. japonicum *from 2005 to 2009.** Prevalence/Incidence represents that of the cohort population. The first point (2005) in Prevalence/Incidence represents infection prevalence of the cohort, and the remaining points represent infection incidence of the cohort. The points in Household (Moran’s I) represent the Moran’s I values at household level.

The infection prevalence of the cohort population decreased from 9.3% in 2005 to 3.7% in 2009, reflecting a 60.2% reduction. By mean of GLM test, this reduction was clear after adjusting for age and gender (Wald *χ*^2^ = 37.81, *P* = 0.000). The positive individual was found in every natural village in both 2005 and 2008. No infections were found in 1 (in both 2006 and 2009) or 2 (in 2007) natural villages in the other years. There was only one natural village where no positive individuals were found during two surveys. The Moran’s I value for households decreased from 0.083 in 2005 to -0.022 in 2009. There was a significant global spatial autocorrelation only in 2005 (*P* < 0.05, 9999 permutations). No significant global spatial autocorrelation was observed in the other years (*P* > 0.05, 9999 permutations). These results indicated that the spatial distribution of *S. japonicum* infections might change from correlation to dispersion. The trend of the Moran’s I value at the household level is very similar to that of the *schistosoma* infection prevalence (Figure 2).

### Local spatial autocorrelation

**Figure 3:**
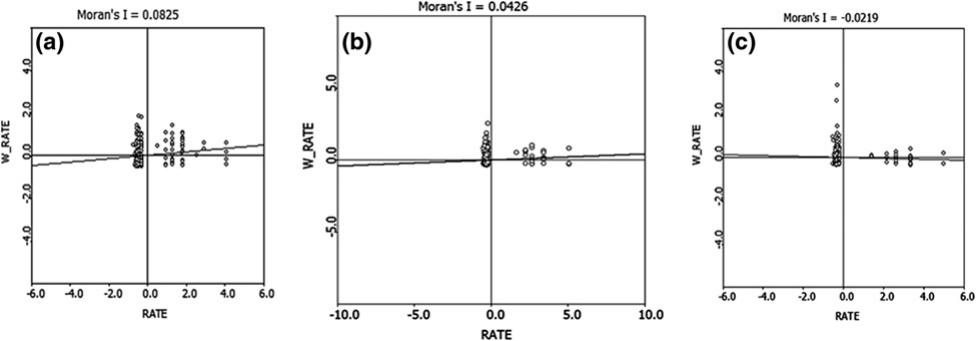
**LISA Moran scatter plot for *S. japonicum *infection prevalence at the household level: (a) 2005, (b) 2007, (c) 2009.**

Based on the values of the global Moran’s I above (i.e. the global Moran’s I value (0.0376) of 2006 is very close to that (0.0426) of 2007 and the global Moran’s I value (-0.0246) of 2008 is very close to that (-0.0219) of 2009) (Figure 2), the data from 2005, 2007, and 2009 were selected to analyze the local spatial autocorrelation of *S. japonicum* transmission. Local Indicators of Spatial Association (LISA) Moran’s I spatial autocorrelation analyses for *S. japonicum* infection prevalence are shown in Figure 3, in which the horizontal axis is the standardized value for *S. japonicum* infection prevalence, and the vertical axis is the mean standardized neighbor value (W_RATE). These Moran scatter plots provide a visual representation of spatial autocorrelation. These plots were divided into four quadrants to allow a qualitative assessment of the association by type: high-high (upper right) and low-low (lower left) as positive local spatial autocorrelation and spatial clusters, and high-low (lower right) and low-high (upper left) as negative correlation and spatial outliers.

Most of the households observed were distributed in the low-high quadrant for the three years examined, and the number of the households in high-high quadrant decreased each year, indicating the spatial distribution of *S. japonicum* infections became less clustered with time(Figure 3). The proportion of significant clusters or outliers in the observed households decreased each year (*P* < 0.05), except for the high-low outliers (*χ*^*2*^ = 1.57, *P* > 0.05). No significant high-high clusters were observed in 2009 (Table 2).

**Table 2:** **Proportion of significant clusters or outliers in the observed households at *α*** **= 0.05 level, 9999 permutations**

**Scatter plot quadrant**	**Year (%)**			***Χ***^***2***^	***P *value**
	**2005**	**2007**	**2009**		
High-high	2.55 (7/275)	0.73 (2/275)	0.00 (0/268)	8.81	0.013
High-low	1.09 (3/275)	0.36 (1/275)	0.37 (1/268)	1.57	0.456
Low-low	19.27 (53/275)	10.55 (29/275)	1.49 (4/268)	45.61	0.000
Low-high	8.36 (23/275)	7.27 (20/275)	3.36 (9/268)	6.30	0.043

The number of significant clusters (or outliers) and observed households are presented in parentheses.

### Space cluster analysis

**Figure 4:**
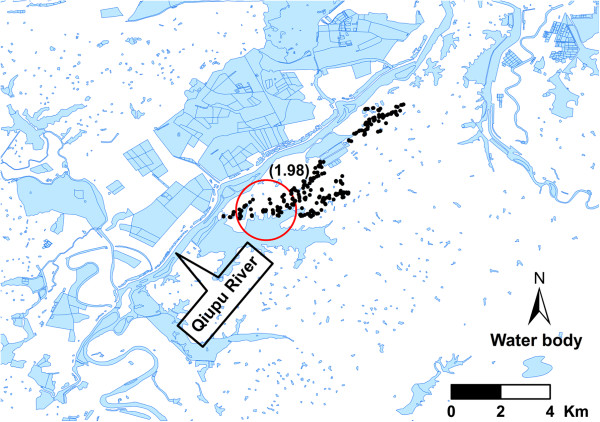
**Spatial cluster location of *S. japonicum *transmission at the household level in 2005 year.** Black dots represent the households. Rate Ratio is presented in the parenthesis.

Only one significant spatial cluster of infection was observed in 2005 (*P* < 0.01) (Figure 4), no significant spatial clusters were found in the other years (*P* > 0.05). The GPS coordinates of the center of the significant spatial cluster was 30.57686 N and 117.37817 E, and the radius of the cluster was 1.44 Km. This cluster included 36 cases and the infection prevalence of the population inside this cluster was 18.5%, while outside the cluster, there were only 25 cases with 5.4% infection prevalence. The cluster was near the Qiupu River (Figure 4).

## Discussion

Transmission of *S. japonicum* is influenced by many factors. Some key factors are climatic suitability (e.g. temperature and precipitation); spatial and temporal distribution of the intermediate host, *O. hupensis*; human activities (e.g. occupational, domestic, and recreational water-contact patterns); environmental contamination with human and animal excreta, in particular, cattle stool; and lack of a clean water supply, sanitation, and proper hygiene [12,24-28]. As a result, *S. japonicum* infection exhibits marked spatial heterogeneity from the community scale even at the scale of a single administrative village) up to the regional scale, [7,17,28-31]. Our results from the spatial autocorrelation analysis showed that there was significant spatial cluster at the household level in 2005, when the infection prevalence was relatively high (i.e. > 9.3%). Similar results were obtained from analyses of the spatial scan statistic that was used to identify areas and population with high prevalence (a significant spatial cluster of infection was found at the household level in 2005). These findings were similar to the results reported by Peng et al. (2010) [17], where the human infection prevalence was 6.5%. Additionally, the results support the general view of the focal nature of *S. japonicum* (i.e. *S. japonicum* infection shows distinct spatial distribution (focality and aggregation) when the human infection prevalence is relatively high).

However, the four years’ results of both the global spatial autocorrelation analysis and the spatial scan statistical analysis all showed that no significant spatial cluster of infection was found at the household level after 2005, the results of the local spatial autocorrelation analysis also showed that the spatial distribution of *S. japonicum* infections became less clustered with time. These suggested that the spatial distribution pattern of *S. japonicum* infections might be changing over the course of control, from a heterogeneous pattern to a more homogenous pattern with a decrease in the infection prevalence at the household scale in our study area. This was different from the general phenomenon of infectious diseases (i.e. spatial heterogeneity increases with declining rates of transmission). This difference might be attributed to or related to 1) the complex life cycle of *S. japonicum* involving snail (*Oncomelania hupensis*) intermediate hosts and many vertebrate definitive hosts [24,32] (for example, the geographic distribution of schistosomiasis japonica largely depends on the availability of the susceptible schistosome-transmitting snail *O.hupensis*[24]*, *and the spatial distribution of snails infected with *S. japonicum* becomes less heterogeneous with a decrease in the infection prevalence [33]); 2) the effective integrated national intervention being employed during the study period [9]; and 3) changes in some important risk factors influencing the transmission of *S. japonicum* in our study area. Although some control measures of snails were carried out in our study region, the spatial distribution of snails does not obviously change, in other words, the area with snails does not change over the course of control [19]. However, the integrated national control strategy resulted in changes in some important risk factors influencing the transmission of *S. japonicum* in the study area [19]. For example, environmental contamination with cattle excreta was completely wiped out after all bovines were removed from the study region. Previously, buffaloes were responsible for approximately 75% of human transmission [34]. Hygiene was improved, to a large extent, by constructing lavatories and latrines and by supplying tap water. Furthermore, new human infections of schistosomiaisis each year were effectively cleared by treatment with praziquantel. Also, health education programs might have changed human behavior. However, it is unclear whether there would be changes in other risk factors with the shift from clustered to random spatial distribution. For example, whether some minor and neglected risk factors (e.g. environmental contamination with wild (e.g. mouse and rabbit) and other domesticated (e.g. pig and dog) animals excreta and population movement) would become important risk factors of *S. japonicum* transmission when other key risk factors noted above were controlled. This is necessary to further survey and to reassess the risk factors of *S. japonicum* transmission. The findings of random spatial distribution patterns of *S. japonicum* transmission showed that it might be necessary to adjust current control measures in the community due to the changes in risk factors of *S. japonicum* transmission. For example, if some neglected risk factors (e.g. wild mouse, population movement) become important risk factorsfor *S. japonicum* transmission in a community after bovines are removed as important sources of infection, an approach for wild rat control or systematic administration of antischistosomal drugs (e.g. praziquantel) to migrantsmay need to be conducted in the community. This can help to tailor effective, locally adapted control measures [27].

Utzinger et al. (2003) [27] reported that the spatial distribution of *S. mansoni* infection intensity levels was random or homogenous in a single village, but the infection prevalence of *S. mansoni* was very high (80.4%) when compared with the infection prevalences of *S. japonicum* in our study. In our study, we saw a random spatial pattern of *S. japonicum* infection only when the human infection prevalence was relatively low (< 4%); when the infection prevalence was relatively high (e.g. > 9%), the spatial pattern was heterogeneous. These findings indicate that spatial patterns of *Schistosoma* transmission might be relevant to infection prevalence at a finer scale, but it is unclear whether there is a critical prevalence when the distribution shifts from heterogenous to homogenous distribution. Additionally, these results suggest that differences in spatial distribution patterns of schistosomiasis might be related to differences in the spatial distribution of risk factors of *Schistosoma* transmission, this needs further study.

The cohort population was a stable population except in 2008 when 8.8% of the participants were lost to follow-up. This was mainly due to population movement (e.g. mobility for paid work or school). There was no obvious difference (e.g. age, sex, occupation) between the observed population and the population lost to follow-up (data not shown). A recognized limitation of this study was related to the Kato-Katz thick smear technique used. This method has become relatively insensitive due to widespread chemotherapy that results in generally low worm burdens [35]; however, our previous study [36] showed that the sensitivity of a single stool examination with the three-slide Kato-Katz method did not change significantly when the prevalence of a single stool examination with the three-slide Kato-Katz method is between 13.0% (close to the baseline (9.3%) in 2005) and 3.9% (close to the prevalence of 3.7% in 2009). Significant spatial clusters of infection could be found by both the spatial autocorrelation analysis and the spatial scan statistical analysis in 2005; however, significant spatial clusters of infection could not be found after 2005. So, the relative insensitivity of Kato-Katz method might not be a major factor for the apparent lack of clustering. Other weaknesses of the study include a relatively small dataset and some risk factor data related to households or individuals were not collected (e.g. human activities and hygiene). This limits the ability to further analyze why the spatial distribution of *S. japonicum* infections changed. Given the results of the present study, it is cautiously suggested that, at the household level, the spatial distribution of *S. japonicum* infections become less heterogeneous as the prevalence of infection decreases.

## Conclusions

The spatial distribution of *S. japonicum* infections might become less heterogeneous as the prevalence of infection decreases at a household scale in our study field. The risk factors of *S. japonicum* transmission might change in our study field. These imply that it may be necessary to re-assess risk factors of *S. japonicum* transmission over the course of control and to adjust control measures accordingly in the communities.

## Competing interests

The authors declare that they have no competing interests.

## Authors’ contributions

YBZ, SL, GMZ, QWJ conceived and designed the study. YBZ, GXC, SMH, ZGH, YPL, JGW implemented the surveys. YBZ, SL, YPL, QWJ analyzed the data. YBZ, CR, SL, QWJ wrote the paper. All authors read and approved the final manuscript.

## Authors’ information

ZYB (PhD) is an associate professor in the Division of Epidemiology, and his primary research interest is the epidemiology and control of Schistosomiasis and GIS. SL (PhD) is an associate professor of GIS and mathematic model. GMZ (PhD) is a professor of epidemiology. QWJ (MD) is a professor of epidemiology. GXC is the director of Guichi Anti-schistosomiasis Station. SMH and ZGH are staff of Guichi Anti-schistosomiasis Station. CR (PhD) and YPL (MD) are students.

## Pre-publication history

The pre-publication history for this paper can be accessed here:

http://www.biomedcentral.com/1471-2458/13/297/prepub

## References

[B1] King CH Dickman K Tisch DJ Reassessment of the cost of chronic helmintic infection: a meta-analysis of disability related outcomes in endemic schistosomiasis Lancet 2005 365 1561 1569 10.1016/S0140-6736(05)66457-4 15866310

[B2] Gryseels B Polman K Clerinx J Kestens L Human schistosomiasis Lancet 2006 368 1106 1118 10.1016/S0140-6736(06)69440-3 16997665

[B3] Steinmann P Keiser J Bos R Tanner M Utzinger J Schistosomiasis and water resources development: systematic review, meta-analysis, and estimates of people at risk Lancet Infect Dis 2006 6 411 425 10.1016/S1473-3099(06)70521-7 16790382

[B4] Caffrey CR Chemotherapy of schistosomiasis: present and future Curr Opin Chem Biol 2007 11 433 439 10.1016/j.cbpa.2007.05.031 17652008

[B5] Zhou XN Wang LY Chen MG Wu XH Jiang QW Chen XY Zheng J Utzinger J The public health significance and control of schistosomiasis — then and now Acta Trop 2005 96 97 105 10.1016/j.actatropica.2005.07.005 16125655

[B6] Chen MG Feng Z Schistosomiasis control in China Parasitol Int 1999 48 11 19 10.1016/S1383-5769(99)00004-5 11269321

[B7] Utzinger J Zhou XN Chen MG Bergquist R Conquering schistosomiasis in China: the long march Acta Trop 2005 96 69 96 16312039 10.1016/j.actatropica.2005.08.004

[B8] Zhou XN Guo JG Wu XH Jiang QW Zheng J Dang H Wang XH Xu J Zhu HQ Wu GL Li YS Xu XJ Chen HG Wang TP Zhu YC Qiu DC Dong XQ Zhao GM Zhang SJ Zhao NQ Xia G Wang LY Zhang SQ Lin DD Chen MG Hao Y Epidemiology of schistosomiasis in the People’s Republic of China, 2004 Emerg Infect Dis 2007 13 1470 1476 10.3201/eid1310.061423 18257989 PMC2851518

[B9] Wang LD Guo JG Wu XH Chen HG Wang TP Zhu SP Zhang ZH Steinmann P Yang GJ Wang SP Wu ZD Wang LY Hao Y Bergquist R Utzinger J Zhou XN China’s new strategy to block Schistosoma japonicum transmission: experiences and impact beyond schistosomiasis Trop Med Int Health 2009 14 1475 1483 10.1111/j.1365-3156.2009.02403.x 19793080

[B10] Clennon JA Mungai PL Muchiri EM King CH Kitron U Spatial and temporal variations in local transmission of *Schistosoma haematobium* in Msambweni, Kenya Am J Trop Med Hyg 2006 75 1034 1041 17172362

[B11] Clennon JA King CH Muchiri EM Kariuki HC Ouma JH Mungai P Kitron U Spatial patterns of urinary schistosomiasis infection in a highly endemic area of coastal Kenya Am J Trop Med Hyg 2004 70 443 448 15100462

[B12] Yang GJ Vounatsou P Zhou XN Tanner M Utzinger J A Bayesian-based approach for spatio-temporal modeling of county level prevalence of *Schistosoma japonicum* infection in Jiangsu province, China Int J Parasitol 2005 35 155 162 10.1016/j.ijpara.2004.11.002 15710436

[B13] Brooker S Beasley M Ndinaromtan M Madjiouroum EM Baboguel M Djenguinabe E Hay SI Bundy DA Use of remote sensing and a geographical information system in a national helminth control programme in Chad Bull World Health Organ 2002 80 783 789 12471398 PMC2567660

[B14] Assunção RM Reis EA A new proposal to adjust Moran’s I for population density Stat Med 1999 18 2147 2161 10.1002/(SICI)1097-0258(19990830)18:16<2147::AID-SIM179>3.0.CO;2-I 10441770

[B15] Kulldorff M Spatial scan statistic Commun Statist - Theory Meth 1997 26 1481 1496 10.1080/03610929708831995

[B16] Kulldorff M Nagarwalla N Spatial disease clusters: detection and inference Stat Med 1995 14 799 810 10.1002/sim.4780140809 7644860

[B17] Peng WX Tao B Clements A Jiang QL Zhang ZJ Zhou YB Jiang QW Identifying high-risk areas of schistosomiasis and associated risk factors in the Poyang Lake region, China Parasitology 2010 137 1099 1107 10.1017/S003118200999206X 20128946

[B18] Zhang ZJ Carpenter TE Lynn HS Chen Y Bivand R Clark AB Hui FM Peng WX Zhou YB Zhao GM Jiang QW Location of active transmission sites of *Schistosoma japonicum* in lake and marshland regions in China Parasitology 2009 136 737 746 10.1017/S0031182009005885 19416552

[B19] Zhou YB Liang S Chen GX Rea C He ZG Zhang ZJ Wei JG Zhao GM Jiang QW An integrated strategy for transmission control of *Schistosoma japonicum* in a marshland area of China: findings from a five-year longitudinal survey and mathematical modeling Am J Trop Med Hyg 2011 85 83 88 10.4269/ajtmh.2011.10-0574 21734130 PMC3122349

[B20] Katz N Chaves A Pellegrino J A simple device for quantitative stool thick-smear technique in Schistosomiasis mansoni Rev Inst Med Trop Sao Paulo 1972 14 397 400 4675644

[B21] Guo J Li Y Gray D Ning A Hu G Chen H Davis GM Sleigh AC Feng Z McManus DP Williams GM A drug-based intervention study on the importance of buffaloes for human *Schistosoma japonicum* infection around Poyang Lake, People’s Republic of China Am J Trop Med Hyg 2006 74 335 341 16474093

[B22] McCullagh P Nelder JA Generalized linear models 1989 second London: Chapman and Hall

[B23] Dwass M Modified randomization tests for nonparametric hypotheses Ann Math Stat 1957 28 181 187 10.1214/aoms/1177707045

[B24] Zhou YB Zhuang JL Yang MX Zhang ZJ Wei JG Zhao GM Zhao GM Zhang SM Jiang QW Effect of low temperature on the schistosome-transmitting snail *Oncomelania hupensis* and the implications of global climate change Molluscan research 2010 30 102 108

[B25] Zhou XN Yang GJ Yang K Wang XH Hong QB Sun LP Malone JB Kristensen TK Bergquist NR Utzinger J Potential impact of climate change on schistosomiasis transmission in China Am J Trop Med Hyg 2008 78 188 194 18256410

[B26] Huang YX Manderson L The social and economic context and determinants of schistosomiasis japonica Acta Trop 2005 96 223 231 10.1016/j.actatropica.2005.07.015 16202596

[B27] Utzinger J Muller I Vounatsou P Singer BH N’Goran EK Tanner M Random spatial distribution of *Schistosoma mansoni* and hookworm infections among school children within a single village J Parasitol 2003 89 686 692 10.1645/GE-75R 14533674

[B28] Kloos H Gazzinelli A Van Zuyle P Microgeographical patterns of schistosomiasis and water contact behavior; examples from Africa and Brazil Mem Inst Oswaldo Cruz 1998 93 Suppl 1 37 50 9921322 10.1590/s0074-02761998000700006

[B29] Hotez PJ Zheng F Long-qi X Ming-gang C Shu-hua X Shu-xian L Blair D McManus DP Davis GM Emerging and reemerging helminthiases and the public health of China Emerg Infect Dis 1997 3 303 310 10.3201/eid0303.970306 9284374 PMC2627641

[B30] Mott KE Nuttall I Desjeux P Cattand P New geographical approaches to control of some parasitic zoonoses Bull World Health Organ 1995 73 247 257 7743598 PMC2486751

[B31] Woolhouse ME Chandiwana SK Spatial and temporal heterogeneity in the population dynamics of Bulinus globosus and Biomphalaria pfeifferi and in the epidemiology of their infection with schistosomes Parasitology 1989 98 Pt 1 21 34 2717216 10.1017/s0031182000059655

[B32] Zhou YB Liang S Jiang QW Factors impacting on progress towards elimination of transmission of schistosomiasis japonica in China Parasites & Vectors 2012 5 275 10.1186/1756-3305-5-275 23206326 PMC3519747

[B33] Yao BD Zhou YB Wang ZL Tian AP Zhu SP Wei CJ Yang QY Lu BK Liao YZ Hu BJ Yi P Jiang QW Study on spatial-temporal variation of infected snail in bottomland areas after an integrated control strategy at village level in the marshland and lake regions based on geographic information system Zhonghua Liu Xing Bing Xue Za Zhi 2012 33 702 705 22968020

[B34] Gray DJ Williams GM Li Y McManus DP Transmission dynamics of *Schistosoma japonicum* in the lakes and marshlands of China PLoS One 2008 3 e4058 10.1371/journal.pone.0004058 19115007 PMC2605259

[B35] Zhou YB Zheng HM Jiang QW A diagnostic challenge for schistosomiasis japonica in China: consequences on praziquantel-based morbidity control Parasites & Vectors 2011 4 194 10.1186/1756-3305-4-194 21981948 PMC3195757

[B36] Zhou YB Yang MX Wang QZ Zhao GM Wei JG Zhao GM Jiang QW Field comparison of immunodiagnostic and parasitological techniques for the detection of schistosomiasis japonica in the people’s republic of China Am J Trop Med Hyg 2007 76 1138 1143 17556625

